# Selective reconstitution of liver cholesterol biosynthesis promotes lung maturation but does not prevent neonatal lethality in Dhcr7 null mice

**DOI:** 10.1186/1471-213X-7-27

**Published:** 2007-04-04

**Authors:** Hongwei Yu, Man Li, G Stephen Tint, Jianliang Chen, Guorong Xu, Shailendra B Patel

**Affiliations:** 1 Division of Endocrinology, Metabolism and Nutrition, Medical College of Wisconsin, Milwaukee, WI 53226, USA; 2 Research Service, Department of Veterans Affairs New Jersey Health Care System, East Orange, NJ 07018, USA; 3 Department of Medicine, UMDNJ-New Jersey Medical School, Newark, NJ 07103-2714, USA; 4 Department of Veterans Affairs, Clement J. Zablocki Medical Center, Milwaukee, WI 53295, USA; 5 Qilu Hospital of Shandong University, 44 West Wenhua Road Jinan, 250012, P. R. China

## Abstract

**Background:**

Targeted disruption of the murine 3β-hydroxysterol-Δ7-reductase gene (*Dhcr7*), an animal model of Smith-Lemli-Opitz syndrome, leads to loss of cholesterol synthesis and neonatal death that can be partially rescued by transgenic replacement of DHCR7 expression in brain during embryogenesis. To gain further insight into the role of non-brain tissue cholesterol deficiency in the pathophysiology, we tested whether the lethal phenotype could be abrogated by selective transgenic complementation with DHCR7 expression in the liver.

**Results:**

We generated mice that carried a liver-specific human *DHCR7 *transgene whose expression was driven by the human apolipoprotein E (ApoE) promoter and its associated liver-specific enhancer. These mice were then crossed with *Dhcr7+/- *mutants to generate *Dhcr7-/- *mice bearing a human *DHCR7 *transgene. Robust hepatic transgene expression resulted in significant improvement of cholesterol homeostasis with cholesterol concentrations increasing to 80~90 % of normal levels in liver and lung. Significantly, cholesterol deficiency in brain was not altered. Although late gestational lung sacculation defect reported previously was significantly improved, there was no parallel increase in postnatal survival in the transgenic mutant mice.

**Conclusion:**

The reconstitution of DHCR7 function selectively in liver induced a significant improvement of cholesterol homeostasis in non-brain tissues, but failed to rescue the neonatal lethality of Dhcr7 null mice. These results provided further evidence that CNS defects caused by Dhcr7 null likely play a major role in the lethal pathogenesis of *Dhcr7*^-/- ^mice, with the peripheral organs contributing the morbidity.

## Background

The role of cholesterol in embryonic development is an important question in biology, with significant ramifications for human disease [1,2]. Defects in post-squalene cholesterol biosynthesis, such as in Smith-Lemli-Opitz syndrome (SLOS, MIM 270400) or desmosterolosis (MIM 603398), disrupt the synthesis of cholesterol and cause a variety of severe developmental abnormalities [3-8]. SLOS is a complex inborn error of cholesterol biosynthesis caused by mutations of the 3β-hydroxysterol-Δ7 reductase gene (*DHCR7*) [9-11]. The lack of *Dhcr7 *expression in mouse mimics the early postnatal lethality observed in severely affected individuals (those with the condition formerly referred to as SLOS type II). The biological changes in development caused by disruption of normal cholesterol biosynthesis that result in this early postnatal lethality remain obscure. As a genetic model for understanding of the SLOS in human, mice lacking Dhcr7 provide a useful model to determine which affected tissues are critically responsible for the lethality and which ones may contribute to the morbidity.

One cause of early postnatal death in the Dhcr7 null animals appears to be associated with anoxia due to diffuse atelectasis of the late gestational lungs [12-14], which may cause immature formation of gas-exchange unit and subsequent respiratory insufficiency after birth [2,14]. Therefore, lung involvement seems to be a cause of neonatal death in Dhcr7 null mouse model. However, there is still uncertainty regarding the mechanism(s) responsible for the late gestational lung hypoplasia caused by the loss of endogenous cholesterol biosynthesis in *Dhcr7*^-/- ^mice. As with any biological aspect of a generalized cholesterol deficiency, the pathophysiology is likely to be complex and multi-factorial. It remains unclear whether the delayed lung maturation is caused by a developmental defect intrinsic to lung or whether it represents a systemic abnormality, especially of the central nervous system (CNS), because of impaired cholesterol homeostasis during embryogenesis. A different variety of developmental abnormalities in brain have also been noted in both human patients and, to some extent, in Dhcr7-deficient animal models. In this regard, we have also recently demonstrated that *Dhcr7*^-/- ^mice can be partially rescued from neonatal death by a low level restoration of DHCR7 expression in brain, indicating that neuropathophysiology is one cause of their neonatal lethality [15].

In attempting to rescue *Dhcr7*-deficient mice, we felt that it was necessary to consider the liver because that organ is a major source of cholesterol for most developing organs, except for brain [4,16-19]. Thus, we hypothesized that restoration of normal cholesterol synthesis in the liver might be able to abrogate the lethality. In the current study, we asked whether the specific reconstitution of DHCR7 expression in liver would alleviate the cholesterol deficiency and afford protection from early postnatal death in Dhcr7 null mice. Our results suggest that, although expression of DHCR7 in liver alone during development induced a significant improvement in cholesterol homeostasis in non-brain tissues and promoted lung maturation, it failed to rescue the neonatal lethality. These results provided further evidence that CNS defects caused by Dhcr7 null likely play a major role in the lethal pathogenesis of *Dhcr7*^-/- ^mice, with the peripheral organs contributing the morbidity.

## Results

### Characterization of TgDHCR7 lines

**Figure 1:**
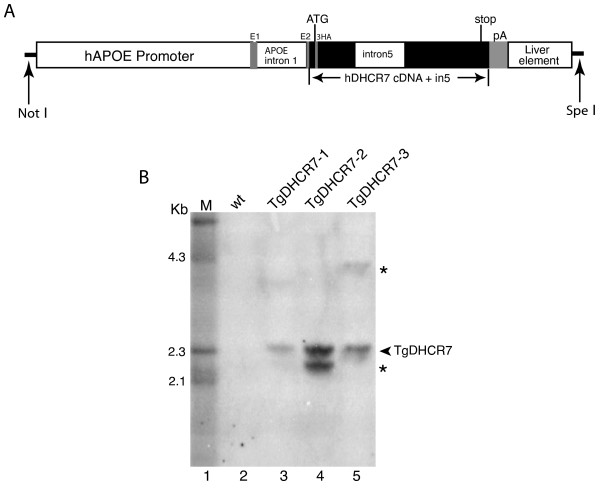
**Generation of *TgDHCR7 *mice**. **Panel A **shows a cartoon of the transgene construct used for microinjection. The human Apo E promoter containing non-coding exon 1 (E1), intron 1 and part of non-coding exon 2 (E2) was fused to a N-terminal 3HA tagged human DHCR7 cDNA containing intron 5, followed by a human Apo E poly A addition site (pA), and a liver enhancer element. **Panel B **shows the results of the Southern blot analysis of *Bam *HI-digested DNA from the three *TgDHCR7 *(*Tg*) founder mice (lane 3, 4 and 5). Lane 1 is λ-Hind III DNA marker and lane 2 contains DNA from a *Tg *negative control animal. The arrowhead points to the expected size bands for *TgDHCR7 *(2.3 kb). Additional bands (asterisks) indicate the integrated transgene that has undergone some deletions or rearrangements.

**Figure 2:**
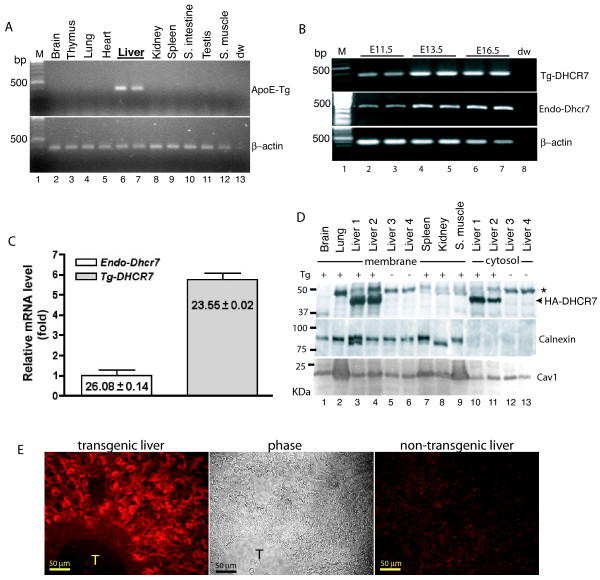
**Characterization of *TgDHCR7 *transgene expression**. **Panel A **shows the tissue distribution of *TgDHCR7 *transgene mRNA as determined by RT-PCR. Amplification was performed with primers bounding the intron in the transgene. Note that expression was readily detectable on the liver tissues only. **Panel B **shows the profile of *TgDHCR7 *transgene expression during embryonic liver development, determined by RT-PCR. The samples were taken from embryonic days as indicated. Robust expression was detectable in liver tissue as early as E11.5. **Panel C **shows quantitation of transgene mRNA expression (*TgDHCR7*) relative to endogenous Dhcr7 (*endo-Dhcr7*) in liver samples by real-time RT-PCR. The transgene message was almost 5-fold greater than endogenous *Dhcr7 *message. The mean ± SD of Ct values of *Tg-DHCR7 *and endogenous *Dhcr7 *are shown in chart. **Panel D **shows protein expression of transgenic HA-tagged DHCR7 from the multiple tissues of *TgDHCR7 *(*Tg*) positive (+) and *Tg *negative (-) control mice. Membrane and cytosolic fractions from the multiple tissues of *TgDHCR7-2 *and *TgDHCR7-3 *positive animals (liver 1 and 2 respectively, lanes 3 and 4) and the livers of *Tg *negative control mice (liver 3 and 4, lanes 5 and 6) were prepared for immunoblotting analyses as described in Methods. The upper panel shows western blotting data using monoclonal anti-HA antibody (arrow denotes DHCR7 ~43 kDa). The asterisk at 48 KDa indicates immunoreactivity to IgG heavy chain in the samples. Middle and lower panels are immunoblots for calnexin (an ER membrane marker) and caveolin-1 (cav1, a plasma membrane marker), respectively, as controls. **Panel E **shows immunohistochemical staining with anti-HA antibody (left panel) and phase-contrast (middle panel) images of transgenic liver sections. No signal was detected with the antibody in control liver (right panel). T indicates liver portal triad.

Three mice (one female and two males), referred as to TgDHCR7-1, TgDHCR7-2 and TgDHCR7-3, respectively (Fig. 1), were identified as transgenic on the basis of PCR and Southern blot analyses of tail samples of genomic DNA. Multiple tissues were collected from N2 transgenic progeny from the founder mice crossed with C57Bl/6J at postnatal day (P) 10 and analyzed for transgene expression by RT-PCR and immunoblotting. Both TgDHCR7-2 and TgDHCR7-3 transgenic lines expressed human DHCR7 mRNA robustly in the postnatal livers, but not in the other tissues (Fig. 2A,C) and western blotting analyses showed expression of HA-tagged protein (Fig. 2D). TgDHCR7 mRNA transcript was detected in transgenic liver at E11.5 (the earliest we could dissect liver tissue) by RT-PCR determination (Fig. 2B) and was quantitatively ~5 fold higher than endogenous murine Dhcr7 mRNA in the transgenic liver at E16.5 as judged by real-time PCR (Fig. 2C). Immunofluorescence staining with anti-HA antibody showed that TgDHCR7 was highly expressed in hepatocytes in liver sections, but such signal was not detectable in control liver (Fig. 2E). Further studies therefore utilized these two lines, which were backcrossed with C57Bl/6J for 4 to 5 generations. Since no differences between either line were identified, these lines were used interchangeably.

### Sterol metabolic profile of TgDHCR7/Dhcr7^-/- ^mice

**Figure 3:**
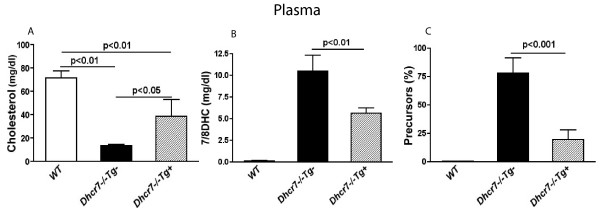
**Pooled plasma sterol analyses from neonatal pups**. Sterol levels in three pooled plasma samples (9 pups per genotype, pooled by combining samples from 3 pups) collected from animals of each genotype. Plasma cholesterol levels (mg/dl) in WT, *Dhcr7*^-/-^*Tg*- and *Dhcr7*^-/-^*Tg*+ neonates were 71.7 ± 5.68, 13.5 ± 1.3 and 33.7 ± 10.4, respectively (**Panel A**). 7/8DHC (mg/dl) was 0.12 ± 0.04 in WT plasma and a massive accumulation of 7/8DHC was found in *Dhcr7*^-/-^*Tg*- (10.5 ± 1.8). 7/8DHC level in *Dhcr7*^-/-^*Tg*+ plasma (5.7 ± 0.7) was ~50 % of *Dhcr7*^-/-^*Tg*- level, but still ~47-fold higher than WT level (**Panel B**). The percent of sterol precursors appeared to be dramatically reduced in *Dhcr7*^-/-^*Tg*+ plasma (19.5 ± 7.6 %), compared to *Dhcr7*^-/-^*Tg*- mice (77.6 ± 13.6 %) (**Panel C**). And total sterols (mg/dl) in *WT*, *Dhcr7*^-/-^*Tg*- and *Dhcr7*^-/-^*Tg*+ neonates were 71.8 ± 5.67, 23.9 ± 2.62 and 39.3 ± 10.1, respectively. Results are expressed as mean ± SD.

To determine the physiological consequences of selective restoration of DHCR7 expression in liver, we bred the transgene onto Dhcr7 null background (see Methods). Steady-state sterol levels in P0-P1 neonates were obtained by sacrificing the litters from the initial crosses, tissues and plasma harvested and genotypes performed on DNA extracted from tails. Cholesterol levels in *Dhcr7*^-/-^*Tg*+ plasma (pooled plasma from 9 animals, pooling performed by combining plasma from 3 pups) were significantly improved compared to non-transgenic Dhcr7-null plasma (Fig. 3A), plasma 7-dehydrocholesterol (7DHC) and 8-dehydrocholesterol (8DHC) sterols were reduced and the percentage precursor levels significantly decreased (cf. Fig. 3B and 3C). Thus the expression of the transgene in the liver led to improvements in the plasma sterol profiles.

**Figure 4:**
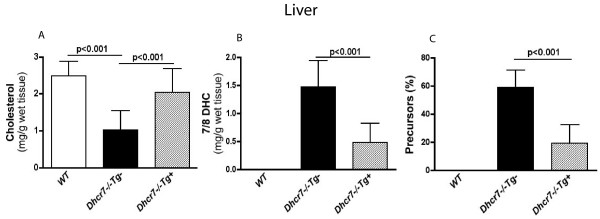
**Sterol analyses of liver tissues from wild-type, knockout and knockout- transgenic pups**. **Panel A **shows that the significant cholesterol deficiency in *Dhcr7*^-/-^*Tg*- neonates (1.0 ± 0.53 mg/g tissue, P < 0.001 *cf *to WT) was restored to normal by the transgene expression (2.03 ± 0.64 mg/g tissue) and was not statistically different from WT livers (2.48 ± 0.38 mg/g tissue). Cholesterol level in livers from *Dhcr7*^+/+^*Tg*+ littermates was 2.45 ± 0.42 mg/g tissue (data not shown). Massive accumulation of 7/8DHC in livers of *Dhcr7*^-/-^*Tg*- neonates (1.46 ± 0.45 mg/g tissue, **Panel B**), was significantly decreased to 0.48 ± 0.33 mg/g tissue in *Dhcr7*^-/-^*Tg*+ livers and only trace levels in livers of WT mice were detected (0.01 ± 0.02 mg/g tissue). The percent of sterol precursors in total sterols was also significantly improved in *Dhcr7*^-/-^*Tg*+ livers (19 ± 13 %), compared to *Dhcr7*^-/-^*Tg*- mice (59 ± 12 %, p < 0.001, **Panel C**). No significant difference (p > 0.05) was found in total sterol levels (mg/g tissue) among the three groups (2.49 ± 0.49 in WT, 2.43 ± 0.53in *Dhcr7*^-/-^*Tg*- and 2.51 ± 0.51in *Dhcr7*^-/-^*Tg*+). Results are expressed as mean ± SD.

Liver sterol profiles in *Dhcr7*^-/-^*Tg*+ pups showed that tissue cholesterol was restored to almost wild-type liver level and precursor levels were reduced by ~70% suggesting the liver defect was not fully restored (Fig. 4). It is important to note that whole liver tissue in the embryonic and neonatal mouse also contains substantial non-hepatic (primarily hematopoetic) cells.

**Figure 5:**
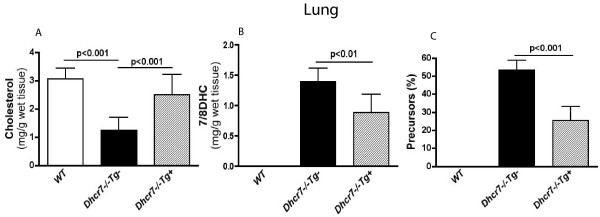
**Sterol analyses of lung tissues from wild-type, knockout and knockout- transgenic pups**. **Panel A **shows that cholesterol level in lung tissues was restored to normal by the liver transgene expression (to 2.51 ± 0.71 mg/g tissue) in *Dhcr7*^-/-^*Tg*+ lungs and was not statistically different from WT lungs (of 3.06 ± 0.40 mg/g tissue), while the non-transgenic-KO lungs exhibited significant cholesterol deficiency (1.25 ± 0.44 mg/g tissue). Cholesterol level in lungs from *Dhcr7*^+/+^*Tg*+ littermates was 3.18 ± 0.54 mg/g tissue (data not shown). **Panel B **shows that this also led to a fall in precursor sterols (0.88 ± 0.30 mg/g tissue in *Dhcr7*^-/-^*Tg*+ *cf *1.39 ± 0.22 mg/g tissue in *Dhcr7*^-/-^*Tg*-), with a significant fall in the percentage of precursor sterol in the lung tissues of the transgenic-KO mice (25 ± 8 %, compared to 53 ± 5 % in *Dhcr7*^-/-^*Tg*- mice, p < 0.001, **Panel C**). There was no significant difference (p > 0.05) in total sterols among the three groups (lung total sterols mg/g tissue: 3.10 ± 0.40 in WT, 2.64 ± 0.62 in *Dhcr7*^-/-^*Tg*- and 3.59 ± 1.08 in *Dhcr7*^-/-^*Tg*+, data not shown). Error bars represent mean ± SD.

**Figure 6:**
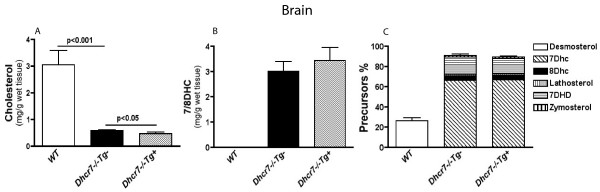
**Sterol analyses in brain tissues from wild-type, knockout and knockout- transgenic pups**. A slightly elevated cholesterol level was found in *Dhcr7*^-/-^*Tg*+ brains, compared to *Dhcr7*^-/-^*Tg*- brains (0.58 ± 0.03 mg/g brain tissue in *Dhcr7*^-/-^*Tg*+ vs. 0.47 ± 0.06 mg/g brain tissue in *Dhcr7*^-/-^*Tg*-, p < 0.05, see Text for discussion) (**Panel A**). However, there were no significant alterations of 7/8DHC levels (3.42 ± 0.53 mg/g tissue in *Dhcr7*^-/-^*Tg*+ vs. 3.01 ± 0.38 mg/g tissue in *Dhcr7*^-/-^*Tg*-, p > 0.05) (**Panel B**), or of the ratios of different precursors in *Dhcr7*^-/-^*Tg+ *(89 ± 1 %), compared to *Dhcr7*^-/-^*Tg*- brains (91 ± 2 %, p > 0.05) (**Panel C**). No significant difference (p > 0.05) was found in brain total sterol levels among three groups (mg/g tissue: 4.63 ± 0.46 in WT, 5.05 ± 0.68 in *Dhcr7*^-/-^*Tg*- and 5.33 ± 0.66 in *Dhcr7*^-/-^*Tg*+, data not shown). Results are expressed as mean ± SD.

Remarkably, tissue sterol levels in *Dhcr7*^-/-^*Tg*+ lung also showed a dramatic restoration of cholesterol (Fig. 5A). Precursor sterol profiles were also significantly improved, and were similar in profile to that observed for the liver (Fig. 5B and 5C), though the reductions were more modest (~40%). In contrast, there was almost no change in the sterol profiles in brain from knockout animals that expressed the transgene in the liver (Fig. 6). Although cholesterol levels in *Dhcr7*^-/-^*Tg*+ brains were statistically higher than non-transgenic mutant brains (Fig 6A), this increase was minimal and the brains showed significant cholesterol deficiency. Additionally, the presence of the transgene had no effect on either the total 7/8DHC precursors (Fig. 6B) or the different species of precursors (Fig. 6C). We speculate that the small increase in brain cholesterol seen in the *Dhcr7*^-/-^*Tg*+ may reflect the contamination of the brain by the increased cholesterol in the blood.

Collectively, these data indicated that selective reconstitution of DHCR7 expression in liver improved significantly cholesterol homeostasis in liver, lung and circulation of Dhcr7 null animals during embryogenesis, but did not affect metabolism in the brain.

### Late gestational lung development in Dhcr7^-/-^Tg+ mice

**Figure 7:**
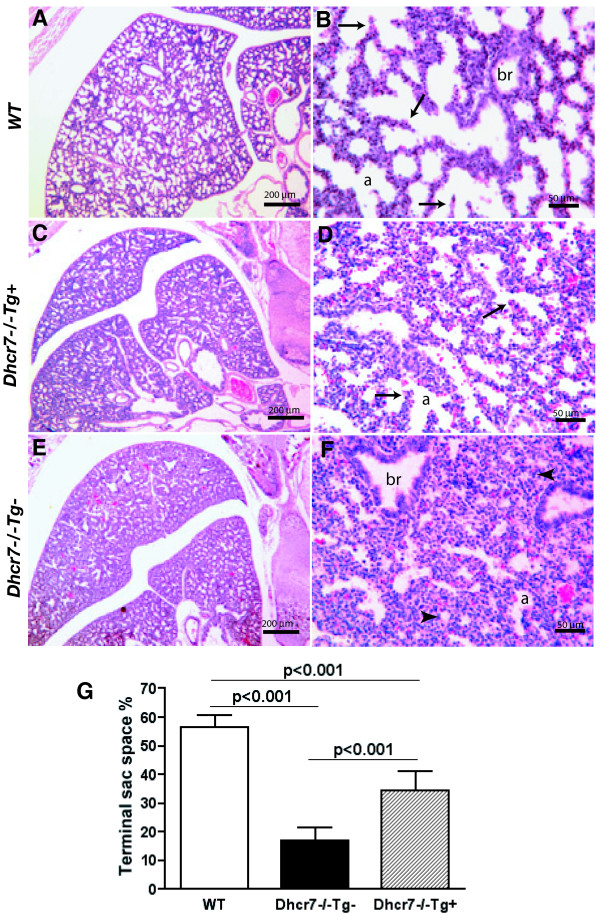
**Lung histology in wild-type, knockout and knockout-transgenic embryos**. Histological sections of lungs from WT, *Dhcr7*^-/-^*Tg+ *and *Dhcr7*^-/-^*Tg- *at term (E19.5) were stained with hematoxylin/eosin (H & E) and photographed. WT lung depicts late saccular phase of lung development (**Panels A and B**). Considerable elongation of the secondary crests (arrows) and a more thinned mesenchyme are evident throughout the WT lung. *Dhcr7*^-/-^*Tg- *lung showed less distal saccular structures, had thick-walled mesenchyme and septation appeared not to be progressing, with undeveloped epithelial tubular structures (arrowheads) throughout the lung fields (**Panels E and F**). Although these changes are also present in the lungs from transgenic-KO lungs, less severe hypoplasic lung phenotypes are evident in *Tg+ *mutant mice (**Panels C and D**, arrows show secondary crests), compared to WT and *Dhcr7*^-/-^*Tg- *animals. **Panel G **shows the morphometric analyses of lung terminal sac spaces in lungs of WT, *Dhcr7*^-/-^*Tg+ *and *Dhcr7*^-/-^*Tg- *at E19.5. Results are expressed as mean ± SD. Labels: a, pre-alveolar spaces; br, bronchiole.

**Figure 8:**
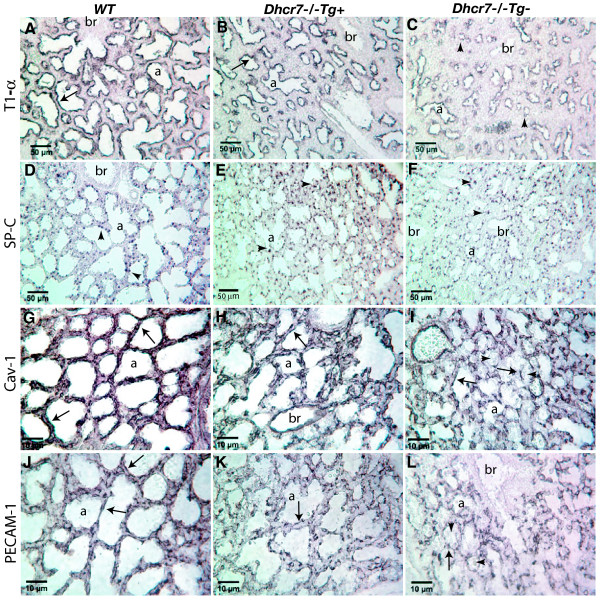
**Immunostaining of selected marker proteins expressed in distal lung**. Lung sections at E19.5 from WT control (**Panels A, D, G and J**), *Dhcr7*^-/-^*Tg+ *(**Panels B, E, H and K**) and *Dhcr7*^-/-^*Tg- *(**Panels C, F, I and L**) were immunostained with antibodies against T1-α, SP-C, caveolin-1 (cav-1), and PECAM-1, respectively. Immunostaining for SP-C shows relative similar patterns and numbers of positive cells (brown dots, indicated by arrowheads) in WT control (Panel D), *Dhcr7*^-/-^*Tg+ *(Panel E) and *Dhcr7*^-/-^*Tg- *(Panel F) lungs. T1-α expression in WT lung (Panel A) is confined to flattened cells lining the pre-alveolar spaces (arrow). A decreased pattern of T1-α expression, especially in undeveloped epithelial tubules (arrowheads), is clearly evident in *Dhcr7*^-/-^*Tg- *lung (Panel C). Both cav-1 (Panel G) and PECAM-1 (Panel J) staining demonstrate an extensive vascular network in the well developed saccules in WT lung, however, delayed vascular development was observed in *Dhcr7*^-/-^*Tg- *lung (Panel I and L). Note that T1-α, cav-1 and PECAM-1 expressive patterns in *Dhcr7*^-/-^*Tg+ *lung (Panels B, H and K, respectively) appear in the middle between WT and *Dhcr7*^-/-^*Tg- *lungs. Labels: a, pre-alveolar spaces; br, bronchiole.

Loss of Dhcr7 in mice leads to subtle, but reproducible defects in lung development; all knockout pups show impaired saccular and capillary growth development caused by developmental arrest at the very last stages of lung development [14]. The primary cell defect seems to be a failure of terminal differentiation of type 1 alveolar epithelial cells (AECs). Since the hepatic transgene expression led to an increase in significant amounts of lung tissue cholesterol, we examined for effects on lung phenotype in near term embryos (E19.5) that were wild-type (WT)*, Dhcr7*^-/-^*Tg*+ and *Dhcr7*^-/-^*Tg*-. At this stage, the lungs of WT mice showed normal sacculation (Fig. 7A) with formation of pre-alveoli that have thin septa (Fig. 7B). In contrast, lungs from Dhcr7 null mice (Fig. 7E and 7F) showed the lack of saccular formation, with failure to thin out the pre-alveolar septae and development of sac spaces, in keeping with our previous findings. Interestingly, the lungs from Dhcr7 null mice expressing DHCR7 in the liver showed an intermediate phenotype, with improved sac space formation and thinning of the pre-alveolar septae (Fig. 7C and 7D). Analyses of the sac space volumes showed that transgenic KO lungs had an increase in terminal sac spaces, compared to non-transgenic mutant lungs (Fig. 7G). Immunohistochemical characterization of these lungs confirmed this histological improvement (Fig. 8). We had previously shown abnormal T1-α, PECAM-1 and caveolin-1 staining patterns in Dhcr7 null lungs, with preserved surfactant protein staining. The transgene expression led to an improvement in all of these staining patterns (Fig. 8, cf. panels B, E, H and K with C, F, I and L), suggesting that transgenic-induced improvement of cholesterol homeostasis in non-brain tissues normalized the architecture of the distal lung sacculation.

### Postnatal survivability of Dhcr7^-/-^Tg+ mice

Since all of the Dhcr7 null pups die within 24 h of birth (usually within 14 h), we wished to see if the improvement in the cholesterol by liver transgene expression improved survival. One hundred and ninety-six pups from *Dhcr7*^+/-^*Tg*+ females bred with *Dhcr7*^+/-^*Tg*+ males were scored for viability. The survival rates, defined as pups remaining alive beyond 24 h after birth, are summarized in Table 1. When observed at birth, in general, pups that appeared bluish, hypotonic, breathed with difficulty, demonstrated no suckling activity, became dehydrated, and died within 24 h after birth were genotyped to be non-transgenic *Dhcr7*^-/-^, all features of being Dhcr7 null, as described previously [14]. Of the *Dhcr7*^-/-^*Tg*+ pups that were relatively pink within minutes after being born, twelve of 28 (42.8%) from TgDHCR7 line-2 and 8 of 14 (57.1%) from TgDHCR7 line-3 survived the first postnatal day, but died within 48 h after birth. *Dhcr7*^-/-^*Tg*+ pups were also observed to show some hypotonia and poor suckling reflexes similar to *Dhcr7*^-/-^*Tg*- pups. The remaining pups were sacrificed after 2 days to confirm that none of the survivors to this point were null for Dhcr7. Therefore, liver-specific restoration of cholesterol biosynthesis improved survival to up to 48 hours, but all Dhcr7 null pups still died. Thus, despite the hepatic, blood and non-brain tissue cholesterol and developmental improvements by hepatic transgene expression, lethality was minimally delayed.

**Table 1:** Survival beyond 24 h of birth in transgenic and non transgenic *Dhcr7-*/pups

	Genotype*	*n*	Survival**		Survival rate (%)
				
			<24 h	>24 h	
*TgDHCR7 *line 2	*WT*	29	0	29	100
	*Dhcr7+/-*	57	0	57	100
	*Dhcr7-/-Tg-*	5	5	0	0
	*Dhcr7-/-Tg+*	28	16	12	42.8
*TgDHCR7 *line 3	*WT*	19	0	19	100
	*Dhcr7+/-*	40	0	40	100
	*Dhcr7-/-Tg-*	4	4	0	0
	*Dhcr7-/-Tg+*	14	6	8	57.1

* *Tg *positive and negative pups are included in *WT *and *Dhcr7+/- *groups. **Survival is defined as being alive beyond 24 hours after being born.

### Association of cholesterol and 7/8DHC with lipid membranes

**Figure 9:**
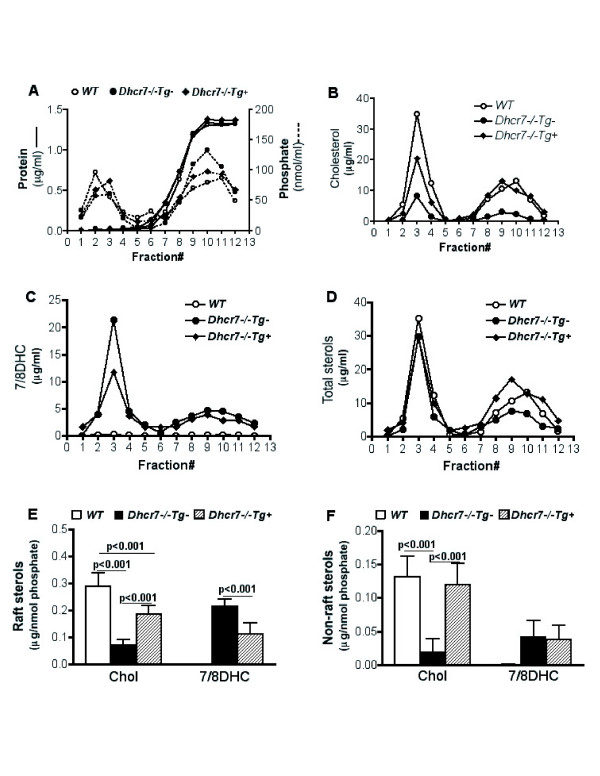
**Characterization of sterol distributions in lipid membranes in wild-type, knockout and knockout-transgenic lungs**. Panel A shows the distributions of protein and phosphate in membrane fractions from sucrose gradients of WT (open circles)*, Dhcr7*^-/-^*Tg- *(closed circles) and *Dhcr7*^-/-^*Tg*+ (filled diamonds) lungs. Fractions 2–4 represent buoyant fractions indicative of rafts. Panel B shows the distribution of cholesterol, Panel C that for precursor sterols 7/8DHC and Panel D that for total sterol (symbols as above). While the total sterol profiles are indistinguishable (Panel D), precursor sterols are increased and cholesterol decreased in the lungs from knockout animals (Panels B and C). Note that the distribution of precursor sterols is also bimodal in knockout lungs and the improvement of the cholesterol/precursor ratio by the Tg status. Quantitative comparison of sterol composition in rafts (fraction 2~4) and non-rafts (fractions 8~11) in *WT, Dhcr7*^-/-^*Tg- *and *Dhcr7*^-/-^*Tg*+ lungs was performed on three separate samples and data were normalized to organic-phosphate, Panel E and F. Results are expressed as mean ± SD. Genotypes are as indicated. See Results for discussion.

Participation in the formation and function of membrane lipid microdomains (rafts) is an important function of cholesterol [20,21]. To address whether the transgene expression led to alterations of sterol distributions between raft and non-raft membrane fractions, we fractionated membranes from lung tissues from these three genotypes. To isolate detergent-resistant membranes (DRMs or rafts), Triton X-100 (TX) extracted postnuclear supernatants prepared from wild-type (WT)*, Dhcr7*^-/-^*Tg- *and *Dhcr7*^-/-^*Tg+ *neonatal lungs were differentially fractionated in discontinuous sucrose gradients [22] and analyzed. Figure 9A showed protein and organic-phosphate distribution in TX sucrose gradients; there were no differences between any of the genotypes. Cholesterol and precursor sterols were analyzed from gradient fractions by GC-MS (Fig. 9B–D). In WT lungs, cholesterol was detected as the only major sterol (Fig. 9B, open circles), 7/8DHC levels were undetectable (Fig. 9C, open circles), and showed a bimodal profile, with one sharp peak corresponding to the raft fractions (fractions 2–4), the second peak found in high-density non-raft fractions (fractions 8~11, Fig. 9B), in keeping with previous reports using detergent or detergent-free methods [23-26]. In *Dhcr7*^-/-^*Tg- *lungs, sterols composed of a mixture of 7/8DHC and cholesterol, also exhibited bimodal profiles, and 7/8DHC constituted the major sterol in both raft and non-raft fractions (Fig. 9B and 9C, filled circles). In *Dhcr7*^-/-^*Tg+ *lungs, although, in both raft and non-raft fractions, cholesterol was dramatically increased, considerable amount of 7/8DHC was still detected (Fig. 9B and 9C, filled diamonds). Total sterol levels (Fig. 9D) were similar in all three groups. Quantitative analyses of sterol content in rafts (n = 3, pooled fractions 2~4) and non-rafts (pooled fractions 8~11) are shown in Fig. 9E and 9F, respectively. Significantly increased cholesterol, ~70 % of WT levels, was observed in rafts from *Dhcr7*^-/-^*Tg+ *lungs, compared to *Dhcr7*^-/-^*Tg- *lungs. However, raft cholesterol in *Dhcr7*^-/-^*Tg+ *lung was still significantly lower than that in WT lungs. Raft 7/8DHC level was significantly reduced, but a considerable accumulation was evident in *Dhcr7*^-/-^*Tg+ *lungs. Interestingly, although non-raft cholesterol levels in *Dhcr7*^-/-^*Tg+ *lungs were comparable to WT, there was no significant decrease of non-raft 7/8DHC levels in *Dhcr7*^-/-^*Tg+ *lungs, compared to *Dhcr7*^-/-^*Tg- *lungs. These data suggest that precursor sterols can be found in raft fractions, but that total sterol distributions appear to be similar in WT and knockout lungs.

## Discussion

Severe cholesterol deficiency, caused by the intentional genetic ablation of *Dhcr7 *in the mouse, has proven to be incompatible with perinatal life and is associated with respiratory insufficiency after birth [12-14]. However, Dhcr7 null pups also exhibit other defects that indicate other organs/systems are also disrupted by absence of normal cholesterol synthesis. A key question is therefore which of these organs (whose lack of normal development) leads to the neonatal lethality. The hypoxia and abnormal lung phenotype would suggest this could be a major defect, though it is also possible that a failure to integrate respiration at the CNS level could be equally important. In our preliminary effort to restore DHCR7 activity in the CNS, we reported a stochastic rescue of pups up to 3 weeks beyond birth, despite almost no increase in brain cholesterol, or fall in precursor sterol concentrations [15]. At the same time, we had also embarked upon restoration of cholesterol synthesis in peripheral (non-brain) tissues to see if this could abrogate the lethality. We report herein the effects of restoration of cholesterol synthesis in the developing liver, an organ that can circulate lipoproteins and cholesterol at a very embryonic age.

Selective reconstitution of liver cholesterol biosynthesis resulted in significant improvement of cholesterol homeostasis in all of the tissues examined in Dhcr7 null mice with the exception of the brain, confirming the embryonic liver's ability to supply cholesterol to peripheral tissues and the inability of cholesterol in the circulation to cross the blood-brain barrier. Cholesterol was detectable in liver and lung of non-transgenic Dhcr7-null term pups at ~40% of normal levels, which represents maternal transfer of this cholesterol and confirmed previous findings [27]. Cholesterol levels in both liver and lungs of transgenic Dhcr7-null term pups increased to ~80% of WT level (and statistically not different from WT levels), indicating robust transgenic liver expression of DHCR7 led to adequate delivery of endogenously synthesized cholesterol to the lungs (presumably via lipoprotein-mediated transfer). The alleviation of the non-brain tissue cholesterol deficit in the transgenic Dhc7-null embryos led to improvement in the lung development, but not complete restoration of normal development.

Although our data support the conclusion that the lung defects may not be the major reason for the neonatal mortality, some caveats need to be mentioned. Despite significantly increased cholesterol in the lung, the sterol precursors were reduced by ~40% and not completely suppressed. Sterol precursors have been demonstrated to be part of plasma membranes and their presence may have significant implications in altering membrane signaling, by altering raft formation and functions [28-33]. In this study, membrane sterol distributions were further investigated and compared between transgenic and non-transgenic *Dhcr7*^-/- ^lungs. Adapting an established method, lipid-rafts and non-rafts were prepared by sucrose gradient fractionation of Triton X100 extracted tissue homogenates [22]. Bimodal profiles of cholesterol and 7/8DHC in sucrose gradient fractions were found, with first peak enriched in "raft" fractions and the second peak found in non-raft fractions. Thus, the bimodal profile may represent sterol portions distributed in lipid-raft microdomains and subcellular membranes [23,25]. As expected, although significant restoration of cholesterol level, a considerable amount of 7/8DHC was still accumulated in the rafts of transgenic *Dhcr7*^-/- ^lungs. Thus one mechanistic possibility is that raft-mediated signaling may be important for the terminal differentiation of Type 1 AECs (and capillary endothelial cells), and that this defect was not fully complemented by increasing the uptake of cholesterol from exogenous sources and thus the lung-associated mortality was not altered. The defect in cholesterol biosynthesis in the lungs will continue to lead to generation of precursor sterols and since these sterols are readily incorporated into plasma membranes, restoration of normal raft function may require direct restoration of Dhcr7 activity [30-34].

Another possibility is that pulmonary surfactant alteration may also play a role in the Dhcr7-deficient lung phenotype, since pulmonary surfactant, the lipid-protein material that stabilizes the respiratory surface of the lung, has been described to function as lipid microdomains. However, we have reported that expression of surfactant proteins, both at the mRNA and at the immunohistochemical level, was indistinguishable between Dhcr7-null and wild-type embryos [14]. Cholesterol concentration, a critical parameter in modulating the lateral structure of pulmonary surfactant membranes as evidenced by in-vitro studies, may provide a structural scaffold for surfactant proteins to act at appropriate local densities and lipid composition [35]. A difference in the condensing ability of cholesterol and 7DHC in monolayer films of dipalmitoyl phosphatidylcholine (DPPC), a major surfactant phospholipid, and their different abilities to form lateral domains with DPPC has also been demonstrated [36]. Sterols can induce upregulation of phosphatidylcholine synthesis in cultured fibroblasts and this process is affected by the double-bond position in the sterol tetracyclic ring structure [37]. Therefore, markedly reduced cholesterol content and the massive accumulation of precursor sterols, such as 7/8DHC, may affect lung developmental at late gestational stages by the modulation of surfactant properties, even if surfactant protein expression is normal. However, restoration of DHCR7 activity in the lung using a transgene that expressed DHCR7 activity did not rescue this lethality (Yu and Patel, unpublished observations). Additionally, our previous brain-specific DHCR7 transgenic expression studies showed that some rescue was possible, even though lung sterol profiles were not altered [15]. Thus, while lung defects remain a contributory factor for this lethality, we contend that a defect in the CNS is the major reason why SLOS pups die soon after birth.

Some positive aspects should also be highlighted. Since the only cells that could synthesize cholesterol were the hepatocytes in the developing liver, yet there was robust restoration of cholesterol in the peripheral organs, this further confirms previous studies on the importance of lipoprotein-mediated liver-derived cholesterol delivery to these organs [18]. Additionally, this study also confirms the now well-established physiological observation that the blood-brain barrier is an absolute barrier for entry of pre-formed cholesterol into the brain and it is operational as early as E10 in the mouse [27].

One curious finding confounding the above interpretations is that, although cholesterol levels in the transgenic Dhcr7-null livers increased to almost WT levels, the precursor levels in the liver were reduced by only 70 %, despite an almost 5-fold increase in the relative expression of the transgene in the liver. Our explanation for this is that the fetal and neonatal mouse liver contains more than hepatocytes, with a significant amount of hematopoetic activity that persists beyond birth. The ApoE promoter chosen will only result in robust hepatic-specific expression. Since total dissected organ tissue sterols are measured, we presume that the precursor accumulations are therefore primarily in these non-hepatic cells and not hepatocytes. If so, it also suggests that close proximity of the hepatocytes and hematopoetic stem cells does not result in complementation or clearance of the precursor sterols from the latter. Thus, while cholesterol can be efficiently delivered to the various tissues readily via the lipoprotein-mediated pathways, removal of the precursor sterols back to the liver may not be as efficient.

## Conclusion

Restoration of liver cholesterol synthesis is an efficient method to restore peripheral tissue cholesterol in Dhcr7-null embryos, and this can lead to an improvement in the lung developmental defects reported previously and a minimal improvement in the neonatal lethality was also observed. However, this strategy does not affect the sterol defect in the CNS and this organ may be critically responsible for the neonatal lethality.

## Methods

### Generation of transgenic mice expressing human DHCR7specifically in liver

To generate transgenic mice expressing DHCR7 in the liver, we used a pLIV-LE6 vector that contains the constitutive human Apo E gene promoter and its hepatic control region (a gift from John Taylor, J. David Gladstone Institutes, San Francisco). N-terminal 3 hemagglutinin (HA) epitope tagged human DHCR7 cDNA with intron 5 (3HA-DHCR7in5) was generated by routine PCR insertion method. The transgenic plasmid (pLiv-11-3HA-DHCR7in5) was generated by cloning 3HA-DHCR7in5 fragment encoding the open reading frame of human DHCR7 into *Mlu *I-*Cla *I sites of pLIV-LE6. The 6-kb *Not *I-*Spe *I fragment of pLiv-11-3HA-DHCR7in5 (Fig. 1) was then isolated and injected into fertilized eggs (C57Bl × FVB/N) to generate transgenic mice by Medical University of South Carolina Transgenic Core facility. Transgene-specific PCR with the forward primer 5'-ATGGAGAGGAGGGGGCTGAGA-3' and the reverse primer 5'-TGGATTTTGCCAATAATGTCCAGC-3' was used for routine genotyping for transgene status. Tail DNA of transgenic (*TgDHCR7*) mice produced a PCR product of 530 bp (data not shown). Southern blots of tail DNAs were performed to identify three founder mice that harbored the integrated transgene. For Southern analysis, 10 μg of DNA was digested with *Bam *HI. The digested DNA was separated on a 0.8% agarose gel, transferred to a Hybond-N membrane, and hybridized with the ^32^P-labeled 1.5 kb human DHCR7 cDNA. Expression levels of the human DHCR7 transgene were determined by RT-PCR and Western blot analyses, on tissues of offspring from positive founders bred with C57BL/6J mice. Quantitative analyses of transgene expression were performed by real-time PCR (see below). Mice with high levels of transgene expression in the liver were bred to C57BL/6J mice, and two integrated transgenic lines, *TgDHCR7-2 *and *TgDHCR7-3*, were established. Both lines showed almost identical patterns of transgene expression and were used interchangeably in all crosses. *Dhcr7*^+/- ^mice (N>12 on C57Bl/6J background), described previously [14,27], were crossed with *TgDHCR7 *lines to generate *Dhcr7*^+/- ^*Tg+ *animals. These were subsequently intercrossed to obtain *Dhcr7*^-/-^*Tg+ *mice. The transgenic mice were maintained as hemizygotes by breeding with C57BL/6J mice (Jackson Laboratories). All mice were housed in colony cages with a 12-hour light/12-hour dark cycle and fed Teklad Mouse/Rat Diet 7002 from Harlan Teklad (Madison, Wisconsin, USA). For animal experiments, non-transgenic littermates were used as controls for transgenic mice. All animal experiments were performed with the approval of the Institutional Animal Care and Research Advisory Committee at Medical College of Wisconsin.

### Tissue expression of TgDHCR7

Total RNA was extracted from multiple tissues from postnatal 10-day-old offspring or livers of embryos at E11.5, E13.5, E16.5 from *TgDHCR7 *lines, as described previously [14,15]. Random hexamers were used as primers for reverse transcription of 1 μg of total RNA, and cDNA first-strand synthesis carried out using Thermoscript (Invitrogen) according to manufacturer's protocol. Specific mRNA for *TgDHCR7 *was amplified using human DHCR7 cDNA primers (forward: 5'-GGACTGGTTTTCACTGGCGAGCG-3' and reverse: 5'-CCAGAGCAGGTGCGTGAGGAG-3'). The endogenous murine Dhcr7 and β-actin were amplified using *Dhcr7 *cDNA primers (forward: 5'-CCAAAGTCAAGAGTCCCAACGG-3' and reverse: 5'-ACCAGAGGATGTGGGTAATGAGC-3') and the β-actin gene primers, as described previously [15]. PCR amplification was carried out as follows: denaturation for 3 min at 94°C and then a succession of 30 cycles by 30 sec at 94°C, 30 sec at 58°C, 1 min at 72°C, and a final extension at 72°C for 10 min. *TgDHCR7 *RT-PCR products (350 bp) were gel-purified and sequenced to confirm it contained correct spliced products using an automated capillary sequencer (Beckman Coulter CEQ 8000, CA), as previously described [15]. SYBR-Green real-time PCR quantification of transgene transcripts was performed with ABI 7300 Real-time PCR system, using human DHCR7 cDNA primers (forward: 5'-CCCAGCTCTATACCTTGTGG-3' and reverse: 5'-CCAGAGCAGGTGCGTGAGGAG-3'), murine Dhcr7 cDNA primers (forward: GCCAAGACACCACCTGTGACAG-3' and reverse: 5'-TGGACGCCTCCCACATAACC-3') and β-actin primers (forward: ACCCTGTGCTGCTCACCGAG-3'and reverse: 5'-TGCCTGTGGTACGACCAGAGG-3'). Quantification of *TgDHCR7 *mRNA levels in transgenic livers on E16.5, relative to endogenous murine Dhcr7 mRNA, was performed using quantitative real-time PCR (qRT-PCR), with normalization performed to the β-actin mRNA abundance from the same sample.

For immunoblotting analysis of TgDHCR7, tissue membrane fractions were extracted using ProteinExtract native membrane protein extraction kit (Calbiochem, San Diego, CA) according to manufacturer's protocol. Briefly, approximately 50 mg of frozen tissues from brain, lung, liver, spleen, kidney, or skeletal muscle was homogenized in 2 ml of ice cold homogenate buffer added with cocktail of protease inhibitors, using a tissue homogenizer (PRO Scientific Inc., Oxford, CT) by three pulses of 10 seconds each and then subjected to Dounce homogenizer, pestle A, by 20 strokes. The homogenates were centrifuged at 16,000 g for 15 minutes at 4°C and supernatants referred as to cytosolic fractions. The pellets were resuspended in lysis buffer and gentled shaken for 30 minutes at 4°C, followed by centrifugation at 16,000 g for 15 minutes at 4°C. The supernatants were collected and referred as to membrane fractions. Protein concentrations in each cytosolic and membrane fractions were determined using Bio-Rad protein assay kit (Bio-Rad, Hercules, CA) and 40 μg of protein from each sample was separated by 8% SDS-PAGE, transferred onto nitrocellulose membranes, incubated with HA monoclonal antibody (1:400, Santa Cruz Biotechnology, Santa Cruz, CA), Calnexin polyclonal antibody (1:200, Santa Cruz Biotechnology), or caveolin-1 (1:400, Santa Cruz Biotechnology) for 2 hours, followed by HRP second antibodies (1:4000, Santa Cruz Biotechnology), and visualized by chemiluminescent detection according to manufacturer's protocols (Perkin-Elmer Life Sciences, Boston, MA).

### Biochemical analyses

Sterol composition in tissues and plasma (pooled plasma collected by decapitation from 9 animals, pooling performed by combining plasma from 3 pups) were identified and quantitated by gas-chromatography-mass spectrometry (GC-MS) as described previously [3].

### Histology and immunohistochemistry

Timed pregnant females at embryonic day (E) 19.5 were sacrificed. E19.5 fetuses were dissected free from the uteri and thoracic body parts harvested, fixed in 10% neutral buffered formalin, and embedded in paraffin. Tissues from non-transgenic *Dhcr7*^+/+ ^*(WT)*, *Dhcr7*^-/- ^*Tg*- and *Dhcr7*^-/- ^*Tg*+ animals were cut into 5-μm sections and stained with hematoxylin-eosin for routine histological examination. Immunohistochemistry (IHC) to determine T1-α, SP-C, caveolin-1 (cav-1) and PECAM-1 expression in tissue sections was performed in standard IHC procedures as described previously [14]. Immunofluorescence staining was performed to determine TgDHCR7 expression in livers by use of a monoclonal anti-HA probe conjugated with TRITC (1:200, Santa Cruz Biotechnology).

### Isolation of detergent-resistant lipid rafts

A modification of the method of Mukherjee et al. [22] was used to isolate lipid rafts from neonatal lung tissues. Briefly, frozen lung tissues were homogenized in an ice-cold lysis buffer containing 5% glycerol in buffer A (50 mM Tris-HCl, pH 8.0, 10 mM MgCl_2_, 0.15 M NaCl, 20 mM NaF, 1 mM Na_3_VO_4_, 5 mM β-mercaptoethanol, 10 μg/ml aprotinin, 10 μg/ml leupeptin, 1 mM PMSF). Tissue debris and nuclei were removed by centrifugation at 1,000 *g *for 5 minutes. Protein concentration of the postnuclear supernatants (PNS) was measured using Protein Reagent (Bio-Rad) and adjusted to 2 mg/ml. 10 % Triton X-100 (TX) was added to 2 ml of PNS to a final concentration of 0.5 % TX followed by a 30 min incubation on ice. The samples were mixed with equal volume of 80 % (w/v) sucrose in buffer A, and then overlaid with 2.0 ml each of 35, 30, 25, and 5 % (w/v) sucrose (all in buffer A). Sucrose gradient was spun at 38,000 rpm in a Sorval 90 ultracentrifuge using TH-641 rotor for 15 hr at 4°C. Twelve fractions of each 1.0 ml were collected from the top to bottom. Sterol composition in each 12 fractions and pooled lipid-raft and non-raft fractions was quantitatively determined as described above and normalized to organic-phosphate as determined by a phosphate assay [38]. Based on cholesterol profile, as well as cellular organelle protein markers and sphingolipid profile (data not shown), lipid rafts or detergent resistant membranes (DRMs) were defined as the membrane materials that floated at the interface of 5% and 25% sucrose in the density range 1.055~1.115 g/ml (fraction 2~4); non-raft materials were collected in the density range 1.130~1.180 g/ml (fraction 8~11).

## Authors' contributions

HY and SBP conceived of the study and designed the experiments. HY, ML, GRX and JLC carried out the experiments. HY. GST and SBP analyzed the data and prepared the manuscript. All authors read and approved the final manuscript.
